# The Use of the Personal Digital Assistant (PDA) Among Personnel and Students in Health Care: A Review

**DOI:** 10.2196/jmir.1038

**Published:** 2008-10-28

**Authors:** Anna M Lindquist, Pauline E Johansson, Göran I Petersson, Britt-Inger Saveman, Gunilla C Nilsson

**Affiliations:** ^3^Department of Surgery and Perioperation ScienceUmeå UniversityUmeåSweden; ^2^School of Human SciencesUniversity of KalmarKalmarSweden; ^1^E-Health InstituteUniversity of KalmarKalmarSweden

**Keywords:** Informatics, medical informatics, computers, handheld, health personnel, students, health occupations, personal digital assistant

## Abstract

**Background:**

Health care personnel need access to updated information anywhere and at any time, and a Personal Digital Assistant (PDA) has the potential to meet these requirements. A PDA is a mobile tool which has been employed widely for various purposes in health care practice, and the level of its use is expected to increase. Loaded with suitable functions and software applications, a PDA might qualify as the tool that personnel and students in health care need. In Sweden today, despite its leadership role in mobile technologies, PDAs are not commonly used, and there is a lack of suitable functions and software applications.

**Objective:**

The aim of the present review was to obtain an overview of existing research on the use of PDAs among personnel and students in health care.

**Methods:**

The literature search included original peer-reviewed research articles written in English and published from 1996 to 2008. All study designs were considered for inclusion. We excluded reviews and studies focusing on the use of PDAs in classroom situations. From March 2006 to the last update in May 2008, we searched PubMed, CINAHL, Cochrane, IngentaConnect, and a local search engine (ELIN@Kalmar). We conducted a content analysis, using Nielsen’s Model of System Acceptability as a theoretical framework in structuring and presenting the results.

**Results:**

From the 900 references initially screened, 172 articles were selected and critically assessed until 48 articles remained. The majority originated in North-America (USA: n=24, Canada: n=11). The categories which emerged from our content analysis coincided to a certain extent to Nielsen’s Model of System Acceptability (social and practical acceptability), including usefulness (utility and usability) subcategories such as learnability, efficiency, errors, and satisfaction. The studies showed that health care personnel and students used PDAs in patient care with varied frequency. Most of the users were physicians. There is some evidence that the use of a PDA in health care settings might improve decision-making, reduce the numbers of medical errors, and enhance learning for both students and professionals, but the evidence is not strong, with most studies being descriptive, and only 6 randomized controlled trials. Several special software programs have been created and tested for PDAs, and a wide range of situations for their use have been reported for different patient groups. Drug and medical information were commonly accessed by PDA users, and the PDA was often viewed as the preferred tool when compared to paper-based documents. Some users regarded the PDA easy to operate, while others found it difficult in the beginning.

**Conclusions:**

This overview of the use of PDAs revealed a positive attitude towards the PDA, which was regarded as a feasible and convenient tool. The possibility of immediate access to medical information has the potential to improve patient care. The PDA seems to be a valuable tool for personnel and students in health care, but there is a need for further intervention studies, randomized controlled trials, action research, and studies with various health care groups in order to identify its appropriate functions and software applications.

## Introduction

The use of modern technology in health care is exploding. Various technological tools are supposed to make health care more effective and secure, to provide appropriate information, and to make it available on a just-in-time basis. Patient security, quality of care, and accessibility to health care are supposed to be improved through the use of technology of various kinds [1]. Access to up-to-date information may be required anywhere and at any time [2], and Information Communication Technology (ICT) is supposed to facilitate decision-making by supporting health care personnel and students [3].

The potential to improve organizations and make them more effective by means of ICT stands in contrast to its limited use. As regards ICT development in Sweden, the National High-Level Group for e-Health [1] has come to an agreement on establishing cooperation nationwide. User-friendly ICT systems aim to provide more time for health care personnel to spend with patients. Today, ICT is used in all areas of health care for various purposes and in various ways, but even more efficient usability is needed. The use of ICT could be facilitated by making it more user-friendly and thus simplifying the daily routines of health care personnel, an objective that could be met by the PDA [1].

The PDA is a very small and portable, handheld computer, which has many more functions than a calculator, and the capacity to store information much like a Personal Computer (PC) [4]. Basic functionality available on most PDAs includes an address book, schedule, calendar, note pad, and e-mail [5]. The PDA is convenient to use in clinical and field situations for quick data management, and the information can be synchronized with a PC [4,6]. By means of a wireless network, information can be exchanged anytime from anywhere to and from a PDA [6], and the network will provide immediate access to all kinds of necessary clinical and administrative data [5]. “PDA” is used as a generic name for all handheld computers in our review.

Previous medical and health care reviews have summarized the research covering the use of PDAs [2,5], including adoption and barriers [7,8]. PDAs have been employed widely in health care practice, and the level of their use is expected to increase. The PDA is mainly a functional tool, but it is also associated with barriers like insufficient security and technical support [8]. Health care professionals need access to information several times a day, and the PDA has the potential to provide this. For the PDA, there are numerous documents and medical software applications available, with a wide variation in quality [5]. A large number of medical students take advantage of the PDA for educational purposes and patient care with great satisfaction [9]. If loaded with suitable functions and software applications, the PDA might meet the need for having access to up-to-date information on a just-in-time basis, thus making the PDA a qualified support tool for personnel and students in health care. In Sweden today, PDAs are not commonly used by personnel and students in health care, and there is a lack of suitable functionality and software applications designed for PDAs. The aim of the present review was to obtain an overview of existing research on the use of PDAs among personnel and students in health care.

## Methods

A literature search was conducted from March to June 2006, followed by a second search in May 2007, and a third in May 2008, using the following search engines and databases: PubMed, CINAHL, Cochrane, IngentaConnect, and a local search engine named (ELIN@Kalmar). The search terms were similar but adapted according to the nomenclature of the specific databases/search engines (Table 1). Further articles were identified from reference lists in the retrieved articles. We included original, peer-reviewed research articles written in English and published from 1996 to 2008. Review articles and studies focusing the use of PDAs in classroom situations were excluded.

**Table 1:** Literature search—search terms and relevant reference titles

Literature search	Search terms	Relevant reference titles
PubMed	Search was done with Medical Subject Headings (MeSH) and with the text words computers handheld, PDA, personal digital assistant, microcomputers, handheld computers, computers, handheld, mini computers, pocket PC and palm pilot, single and combined with nurse, nursing, medicine, physicians, healthcare, healthcare personnel, health personnel or students	193
CINAHL	Search was done with Subject Headings computers-hand-held, computers-portable, microcomputers and health-personnel, nurses, physicians, students, interns-and-residents	163
Cochrane	Search was done with Medical Subject Headings (MeSH) minicomputers, microcomputers including computers-handheld and with the text words handheld-computer, PDA, microcomputer, minicomputer, mobile-device, health, care	56
(ELIN@Kalmar)	Search was done with the text words handheld-computer, mobile-device, minicomputer, microcomputer, PDA, health, care	49
IngentaConnect	Search was done with the text wFords handheld-computer, PD and, health-care	5
Reference lists		5
**Total relevant references (before excluding duplicates)**		**471**
**./. Duplicates**		**135**
**Relevant references (after excluding duplicates) for abstract screening**		**336**
**Included references**		**48**

The selection of articles was performed in several steps. The number of potentially relevant publications identified was over 900 of which 471 seemed relevant and, after excluding 135 duplicates, 336 remained. After reading available abstracts from those 336 references, 164 were excluded as not being relevant (ie, not original, peer-reviewed research articles or not meeting the aim and/or inclusion criteria), and 172 articles remained. After reading 172 full-text articles, 127 were then excluded as not meeting the aim and/or inclusion criteria and not meeting high or medium values in quality assessment (Table 2). The articles were reviewed independently by two of the authors (AL and PJ). Disagreements were resolved and a consensus was obtained. Of the 336 articles primarily found, 48 articles remained, the adequacy of which was checked by two of the authors (BIS and GN). The 48 articles were included in the present review, 43 from the database search and an additional 5 from the reference lists.

**Figure 1:**
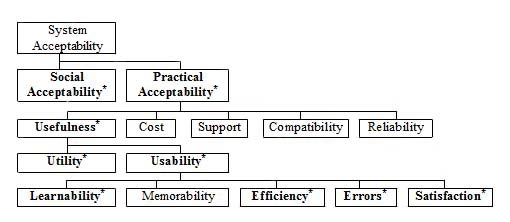
Model of System Acceptability (modified from Nielsen[12])

A content analysis inspired by Burnard [11] was performed and the categories which emerged were: social acceptability, practical acceptability, usefulness, utility, usability, learnability, efficiency, errors, and satisfaction. These categories coincided to a certain extent with Nielsen’s Model of System Acceptability (see Figure 1). The model was used as a theoretical framework in providing a structure to present the results. The remaining categories in Nielsen’s model: system acceptability, cost, support, compatibility, reliability, and memorability were not in agreement with our content analysis and, accordingly, were not used.

**Table 2:** Criteria for quality assessment, based on the criteria for quality assessment from the Swedish Council on Technology Assessment in Health Care (SBU) [10]

Design^*^	I=High	II=Medium	III=Low
RCT	Large and well accomplished multi-center study with sufficient descriptions of protocol, material, and methods. Enough sample size to answer the questions at issue.	neither high nor low	Sample size too small and/or too many interventions to give enough statistical power. Indistinctly described and high participant drop-out rate.
CCT	Well defined questions at issue, sufficient sample size and adequate statistics.	neither high nor low	Small sample size and questionable statistical methods.
DS	Large and well defined consecutive sample analyzed with adequate statistics, long follow-up.	neither high nor low	Small sample size, indistinctly described, follow-up too short, or inadequate statistics.
Q	Well defined questions at issue. Relevant and well described selection, data collection, and analysis. Logically and understandable interpretations and conclusions. Good communicability and conclusions.	neither high nor low	Insufficiently defined questions at issue, selection indistinctly described. Insufficiently described data collection, analysis, interpretations, and conclusions. Indistinct communicability and conclusions.

^*^RCT = randomized controlled trial, CCT=quasi controlled trial, DS=descriptive study, Q=qualitative study.

*System acceptability* is essentially the question of whether the system is good enough to satisfy all the needs and requirements of the users. The acceptability of a computer system is a combination of social and practical acceptability [12]. *Social acceptability* refers to how well a system complies with societal needs such as ethics and legality [12,13]. *Practical acceptability* is determined by usefulness and a number of more traditional attributes such as cost, reliability, and compatibility with existing systems. The *usefulness* category describes whether the system can be used to achieve the desired goals and is further divided into the categories of *utility* and *usability*. Utility refers to whether the functionality of the system can do what is needed, and usability applies to all aspects of a system with which a user may interact, being a question of how well a user can make use of its functionality. Usability has many components and is traditionally divided into 5 key attributes: *learnability*, *efficiency*, *memorability*, *errors,* and *satisfaction*. Learnability implies that the system should be easy to learn and that a user is rapidly able to begin working with the system. If it is efficient, the system should lead to the possibility of high productivity. Memorability in turn means that the system should be easy to remember. The system should have a low error rate and, finally, it should leave users with a feeling of satisfaction [12].

## Results

### Included Articles

The articles included (n=48, see Methods section) were published between 1999 and 2008. They originated from the United States (n=24), Canada (n=11), the United Kingdom (n=4), Hong Kong (n=3), Australia (n=1), Germany (n=1), Norway (n=1), South Korea (n=1), Sweden (n=1), and Taiwan (n=1). A variety of health care personnel and students participated in the studies, mostly physicians and medical students. The research methods varied, with most studies being descriptive and only a few (n = 6) involving randomized controlled trials. The number of participants in the articles varied from 3 to 1185, and the response rate ranged from 24 to 100% (Table 3).

**Table 3:** Articles included in present review (% = response rate)

Authors	Aim	Participants	Methods	Results/conclusions	
Ammenwerth et al (2000) [14] Germany	Evaluate the prototype “a multi-functional mobile information and communication assistant”.	Physicians n=19, nurses n=10, others n=2	One week simulation study, interviews and questionnaires	Participants found needs for mobile computer implementation in clinical routine.	
Aziz et al (2005) [15] UK	Test if a PDA with built-in mobile telephone is more efficient in facilitating communication between health care providers than a hospital pager device.	Surgical physicians n=9	Intervention crossover pilot study, questionnaire	Physicians equipped with a PDA rather than a pager, responded more quickly to a call and had a lower of failure to respond rate.	
Barrett et al (2004) [16] USA	Evaluate PDA use and what advantages and disadvantages a PDA have.	Residents n=88 (40%)	Descriptive study, questionnaire and follow-up interviews	Most residents use PDA daily. The use included commercial medical references and personal organization software.	
Berglund et al (2007) [17] Sweden	Describe nurses and nurse students demands of functions and usability in a PDA	Nurses n=12, nurse students n=84 (75%)	Descriptive study, questionnaire and interviews	With a PDA, the nurses and nurse students expect access to information about the patients, knowledge resources and functions for their daily work.	
Berner et al (2006) [18] UK	Evaluate the effectiveness of a PDA-based clinical decision support system (CDSS) on no steroidal anti-inflammatory drug (NSAID) prescribing safety.	Residents n=68	Randomized controlled trial	Participants provided with a PDA-based CDSS for NSAID prescribing made fewer unsafe treatment decisions than them without.	
Bird & Lane (2006) [19] USA	Determine whether use of a PDA would improve emergency medicine documentation of procedures and patient resuscitations.	Emergency medicine residents (n=35)	PDA procedure were compared with paper-based	Sedation, thoracentesis, and ultrasound documentation significantly increased with PDA vs handwritings.	
Bosma et al (2003) [20] Canada	Assess point-of-care use of PDA in patient consultation management for Intravenous Resource Nurse team (IVRN) consultant service.	Nurses n=5	Intervention study	Team members adopted the new technology with few problems and the service can now efficiently be analyzed.	
Brilla & Wartenberg (2004) [21] USA	Examine the success of intervention of PDAs by comparing PDA use and user attitudes between residents of intervention group and residents in control group.	Neurology residents n=26	Intervention study with control group, structured interviews	Applications most often used were the address book and drug databases. Their use was higher in the intervention group.	
Carroll & Christakis (2004) [22] USA	Determine the percentage of paediatricians using PDAs and computers, as well as the perceived strengths and weaknesses of PDAs.	Paediatricians n=1185 (63%)	Randomized selected descriptive study, questionnaire	35% currently used PDA in work. Most commonly used functions were drug reference, scheduling and medical calculations.	
Chan et al (2004) [23] Hong Kong	Evaluate use of an electronic barcode system in PDA for patient identification during blood transfusion.	41,000 blood samplings	Retrospective study	No incidents of blood transfusion to wrong patients, or wrong labelling of blood samples occurred.	
Chang et al (2004) [24] Taiwan	Develop PDA support systems for mass gatherings and evaluate ease of use and usefulness.	Nurses n=23, physicians n=6	5 simulated Patients’ profiles were tested and evaluated, questionnaire	The PDA system included many information items and was easy to use and useful for mass gatherings.	
Choi et al (2004) [25] South Korea	Evaluate the PDA system MobileNurse.	Nurses n=6	1 day caring for simulated patients was evaluated, questionnaire	Most nurses agreed that MobileNurse was helpful and convenient.	
Criswell & Parchman (2002) [26] USA	Evaluate the uses of handheld computers in family practice residency programs in the United States.	Directors n=306 (50%)	Descriptive study, questionnaire	Two thirds of the education programs used PDAs in their residencies.	
Dee et al (2005) [27] USA	Examined how frequent attending physicians and physicians in training used PDAs for patient care.	Physicians, physicians in training n=108	Descriptive study, questionnaire	87% reported PDA use for patient encounters 55% reported frequent, use for patient care.	
De Groote & Doransk (2004) [28] USA	Determine PDA use on an academic health sciences campus to define the level of training and support the library can provide.	Faculty n=216, medical residents n=124, others n=12 (24%)	Descriptive study, questionnaire	61% of respondents used PDAs. Address book, date book, and calculator were the most commonly used.	
Doran et al (2007) [29] Canada	Develop an electronic information gathering and dissemination system to support both nursing-sensitive outcomes data collection and evidence-based decision-making at the point-of care.	Nurses n=51	Cross-sectional study, work sampling, and focus group interviews	Most priorities were information concerning vital signs, drug information, and manuals of policies and procedures.	
Farrell & Rose (2008) [30] Australia	Investigate whether the use of PDAs enhanced nursing students’ pharmacological knowledge during clinical practice.	Nurse students n=76 (83%)	Quasi-experimental, questionnaire and focus group interviews	PDA users show a higher mean score compared to the control group. The PDA was easy to use and students perceived its use as beneficial to their clinical learning.	
Fischer et al (2002) [31] Canada	Evaluate the feasibility of incorporating handheld computing technology in a surgical residency program.	Residents n=69	Intervention study, questionnaire	After a 5-month pilot period, 38% of surgical residents were using the procedure-logging program successfully.	
Galt et al (2005) [32] USA	Compare drug information sources for PDAs, to minimize medication errors.	General practice physicians n=3	Questionnaire	Lexi-Drugs were found to be the most specific and complete PDA resource.	
Gandsas et al (2004) [33] USA	Compare the ability of surgical residents to identify anatomical structures displayed on a standard monitor versus a PDA screen.	Surgical residents n=23	Randomized cross-over study, questionnaire	The differences between what’s displayed on a standard monitor vs a PDA screen were not significant.	
Garrett & Jackson (2006) [34] Canada	Design, implement, and evaluate a PDA-based e-portfolio tool to support reflective learning in practice.	Nursing students n=6, medical students n=4	Intervention study, questionnaire and focus groups interview	There were positive attitudes to the use of PDA-based tool.	
Goldsworthy et al (2006) [35] Canada	Examine the relationships between the use of PDA and self-efficiency.	Nursing students n=36	Randomized controlled trial	Findings showed a significant increase in self-efficacy in the groups with PDAs.	
Greenfield (2007) [36] USA	Determine whether nursing medication errors could be reduced and nursing care provided more efficiently using PDA technology.	Nurse student n=87 (64%)	Non-randomized quasi-experimental study	Results for accuracy and speed were significantly higher in the PDA group than in the control group.	
Greiver et al (2005) [37] Canada	Explore whether diagnostic software in the PDA would improve care for suspect angina.	Family physicians n=18	Randomized controlled pilot trial	A PDA-based software application can lead to improved care for patients with suspect angina.	
Honeybourne et al (2006) [38] UK	Study impact of PDA on patient care to identify how often and which resources were used, as well as barriers to use in patient care.	Clinical and library staff phase I n=9, phase II n=12	Intervention study, questionnaire	Participants used PDA in clinical setting to support evidence-based practice and education.	
Johnson et al (2004) [39] USA	Describe user acceptance of a suite of programs that deliver information to clinicians’ PDAs.	Faculty, health care personnel n=16	Descriptive study, questionnaire	Most users reported that they learned about new medical developments sooner than they otherwise would have.	
Johnstone et al (2004) [40] Hong Kong	Evaluate the usefulness and acceptability of PDAs loaded with clinical decision software.	Medical students n=169	Randomized controlled trial, questionnaire, and focus group interviews	The students found the PDA useful. They were less satisfied with the functional features.	
Kneebone et al (2003) [41] UK	Describe the use of PDAs in scenario-based clinical procedural skills.	Nursing students, tutors and simulated patients n=25	Evaluation of a PDA-based rating form, observations, and focus group interviews	The PDA forms were easy to use. There were potentially significant advantages over paper-based versions.	
Kushniruk et al (2005) [42] USA	Explore the relationship between system usability and medical errors.	Physicians n=10	Video and audio recorded PDA interactions	Certain types of usability problems were closely associated with the occurrence of specific types of errors in prescription of medications.	
Lapinsky et al (2001) [43] Canada	Evaluate benefits and drawbacks associated with introducing PDA technology in an intensive-care unit.	ICU team with physicians n=20, paramedical staff n=6	Intervention study, scenario tests comparing PDA and paper textbook	PDAs were found to be convenient and functional, but more comprehensive training and improved searching capability were suggested.	
Lau et al (2006) [44] Canada	Understand the current patterns of PDA use among physicians working in palliative medicine.	Physicians n=72	Descriptive study, questionnaire	The PDA was mostly used to organize a practice and look up medical references. Some used it in patient care.	
Leung et al (2003) [45] Hong Kong	Test if a PDA could improve learning in evidence-based medicine.	Medical students n=169	Randomized controlled trial	The PDA improved participants’ educational experience with evidence-based medicine benefiting the most.	
Lu et al (2003) [46] USA	Identify the barriers that impede physicians’ PDA use.	Physicians n=20	Descriptive study, interview	Four barriers were identified: organization, usability, inadequate technology support or access, and lack of need or motivation.	
McAlearney et al (2004) [57] USA	Examine physician’s perspectives about their experiences with PDAs in clinical practice.	Physicians n=54	Qualitative study, focus groups interview	Users seemed generally satisfied, the device helped them increase productivity and improve patient care.
McLeod et al (2003) [47] USA	Investigate PDA use in medical settings, use prevalence, user demo-graphic, and hardware preferences.	Physicians, medical students n=473 (55 %)	Descriptive study, questionnaire	Medical students reported more frequent PDA use in hospital settings and for direct patient care than physicians.	
Mihailidis et al (2006) [48] Canada	Determine what assistive computing device features and functions nurses need.	Nurses n=20	Descriptive pilot study, questionnaire	Data analysis revealed a strong desire to facilitate information access and administer safe medication.	
Morris et al (2007) [49] USA	Understand resident and faculty PDA use and training.	Physicians and n=410 (69%)	Multi-center, questionnaire	Use of PDAs was common. Common barriers were lack of time, knowledge, and formal education.	
Murphy et al (2006) [50] Canada	Determine the frequency of use, usefulness, accessibility, and credibility of PDA, computer, and print drug information resources.	Nurses n=14, physicians n=13 (75%)	Descriptive study, questionnaire	The use of PDAs and computers remains limited. Education for users may facilitate future computer and PDA use.	
Pattillo et al (2007) [51] USA	Identify nursing students’ use of PDAs and compare and contrast the frequency of user resources with comparable text resources.	Nursing students n=90	Intervention study, with control group, questionnaire	The nursing students used their PDAs to look up words and unfamiliar terms, drugs, and the meaning of laboratory values.	
Price (2005) [52] Canada	Examine whether using Palm Prevention improved adherence to 5 preventive measures in primary care.	General practitioners n=8	Randomized controlled trial (pilot study)	The guidelines in PDA increased screening.	
Ranson et al (2007) [53] USA	Understand how physicians use PDAs in their clinical practice and describe how they use a PDA learning portfolio.	Physicians n=10	Literature review and a case study	Information for clinical decisions, patient education and teaching was used and the use was associated with the value of information.
Rothschild et al (2002) [54] USA	Evaluate the clinical contribution of a drug database, usage patterns, decision making etc.	Physicians n=703, medical students n=243 (32%)	Descriptive study, questionnaire	Physicians reported time saving during information retrieval and improves decision making.
Rudkin et al (2006) [55] USA	Assess feasibility of PDA.	Residents n=18, medicine attending n=12	Prospective cross-over time-motion study.	PDAs are feasible in emergency department and change management more often than texts.
Ruland (2002) [56] Norway	Evaluate nurses’ use of CHOICE, a handheld, computer-based support system for preference-based care planning.	Nurses n=28, patients n=155	Intervention study, two control groups	Nurses’ use of CHOICE made nursing care more consistent with patient preferences and improved patients’ preference achievement.
Shiffman et al (1999) [58] USA	Evaluate physician’s satisfaction and frustrations with the use of a PDA based program in asthma care.	Physicians in paediatrics n=9	Descriptive study, questionnaire	Three users gave strongly positive ratings while six users were neutral. Majority used documentation functions.
Stroud et al (2005) [59] USA	Describe the prevalence and patterns of PDA use among nurse practitioners, students, and faculty.	Nurse practitioner students, faculty n=227 (27 %)	Descriptive study, questionnaire	67% of the participants used PDAs. Use was higher among men. Most participants related that PDA use supported clinical decision making.
Teolis et al (2004) [60] USA	Determine what health professionals perceived as barriers to PDA use and how frequently participants used their PDAs for online searching.	Health care personnel n=97, others n=12	Descriptive study, questionnaire and interview	PDAs electronic information and software at point of care, users give users access to a wide variety of also experienced multiple barriers.
Yu et al (2007) [61] USA	Assess the breadth of and determine the patterns of clinical decision support (CDS) program and compare the difference in the recorded and reported PDA CDS utilization among physicians.	Physicians in training n=68 (82%)	A part of a larger study. An automatic tracking program in PDA, questionnaire	Physicians preferred to use certain PDA CDS tools in clinical settings. Drug references and medical calculator were commonly used.

### Users and Situations of Use

The frequency of PDA use varied among different personnel and students in health care [16,21-23,26-28,44,47,59]. Most of the users were male [16,22,59,61], with some exceptions among students [36,47] and faculty [49]. Medical residents used PDAs more than physicians [22,31], but there were also reports of a similar frequency of use amongst the two categories [27], and some physicians used a PDA when teaching medical students [53].

Several special software programs have been created and tested for PDA use. Clinical Decision Support Software (CDSS) has been tested among medical students, and most students agreed that CDSS enhanced their learning, and they became especially fond of their access to Cochrane reviews, history, and physical examination functions [40]. The same decision tool was used by physicians when prescription of pharmaceuticals and safety were evaluated [18]. Physicians using the CDSS for prescription of non-steroidal anti-inflammatory drugs made fewer unsafe treatment decisions than those not using this software. In another study, nurses tested CHOICE, a PDA-based support system for preference-based care planning [56]. The system supported nurses in eliciting patient preferences for functional performance at bedside. Handling CHOICE made nursing care more consistent with patient preferences and improved patients’ preference achievement.

A wide range of situations for use of the PDA have been reported for different patient groups. Guidelines for the management of childhood asthma exacerbations called AsthMonitor were implemented for PDAs and tested in a pilot study [58]. The program supports the documentation of clinical findings and provides guideline-based recommendations. The majority of the physicians in this study frequently applied the documentation functions and found most of the recommendations appropriate. Intelligent, triage-based, mass-gathering emergency medical service PDA support systems were tested among nurses and physicians [24]. The systems included a large number of information items. More than half of the participants perceived that the systems were useful and very easy to use. In another study, nurses used PDA software called MobileNurse which was comprised of 4 different components [25]. The first component was a medical order-checking module, which enables nurses to retrieve patient information, such as physicians’ orders or test results, anywhere and at any time. The second component was a recording module, in which nursing processes at bedside could be recorded. The third component was a nursing unit care plan, and the fourth was a patient information management module by which it was possible to record patients’ demographic information. The participants used the system for 1-day clinical trials, caring for simulated patients. Of those using MobileNurse, 5 of the 6 nurses regarded it to be generally helpful and convenient for checking medical orders and retrieving results of recent clinical tests at bedside [25]. In another pilot study, a software application was tested to help family physicians diagnose angina pectoris among patients with chest pain. This study found that the use of a PDA-based software application for cardiac stress-testing could lead to improved care [37]. For patient identification during a blood transfusion, the addition of an electronic barcode system was made to PDAs [23]. No incidents of blood transfusion to the wrong patients or of the wrong labelling of blood samples occurred with the 41,000 blood-sample procedure carried out during a 3-year period.

### Access to Information

Access to medical reference information and databases is a widely appreciated function of PDA use. Drug and medical information were commonly retrieved by practising PDA users [14-16,19,21,22,24-26,28,30-32,34,35,38-40,43-47,49-51,53-57,59]. Nurses wanted access to drug information, medical references, patient information, medical lists, and test results on a PDA [17,29,48]. In a study of nurses, it was found that 40% of information written on “personal paper” at the point of care was later transcribed to the clinical record. Recording of vital signs and access to reference information about medications on a PDA were top priorities of nurses [29]. Medical students often used drug databases, especially for information about dosage, contraindications, and side effects, but less often for prices [21]. Faculty and health care personnel were presented with headlines about new books, guidelines, reviews, and medical literature on their PDAs [39]. They chose what they were interested in, and the information was delivered to their PDA by their next synchronization. The participants reported that they learned about new medical developments sooner than they otherwise would have and that, without the PDA, they would not have learned about them at all. One intensive-care unit installed a patient-management software program on PDAs, a program including medical reference information, schedules, and contact numbers [43]. Physicians and paramedical staff found the program convenient and functional, especially for patients who had long stays in hospital. An intravenous resource team with a consultant service introduced PDAs for statistical analysis and follow-up evaluation [20].

### Social Acceptability

We identified different barriers to the PDA being socially accepted and to using a PDA at work. Nurses thought it would be a fashionable tool for those most interested in ICT. Some also believed that it would be hard to get acceptance for PDAs among older nurses and nurses that had worked for a long time in a hospital [17]. In another study, PDA use was reported to be a challenge for older physicians [53]. Other nursing students regarded the use of the PDA as rude and inconvenient [30], that the PDA was unnecessary, and that they contributed to a lack of motivation and bad experiences [46,53].

### Practical Acceptability

We found that the PDA was accepted when it solved practical issues. When documents were implemented, the PDA often seemed to be a good tool, preferable to paper-based documents [15,19,41,43,55]. When logged, the PDA-based procedure was preferred and found to be more complete than the handwritten procedure [19]. Similar results were demonstrated when physicians compared electronic medical references [15]. Nursing students and faculty assessing simulated patients found the PDA easy to use when compared to paper work [41]. No difference was noted when text read on a PDA was compared to reading conventional text written on paper [43] and, likewise, when the ability for surgical physicians identifying anatomical structures displayed on a standard monitor was compared to a PDA screen [33]. However, contradictory results have also been reported. Physicians who had previously used a PDA but stopped using it reported reasons like complex and confusing software applications, lack of support, not being useful in practice, cost [44,49], and the inconvenience of carrying it [30,53].

### Usefulness

In the Nielsen model [12], the category of “Usefulness” is divided into the subcategories “Utility” and “Usability” (Tables 4 and 5).

#### Utility

Utility refers to whether the functionality of the PDA can do what is needed [12]. In Table 4 and Table 5 under the subcategory “Utility”, functions and software applications requested and used among personnel and students in health care are presented.

#### Usability

Usability applies to all aspects of a system with which a user may interact and is a question of how well a user can make use of the system’s functionality [12]. In Table 4 and Table 5 under the subcategory “Usability”, functions and software applications evaluated among personnel and students in health care are presented. “Usability” is further divided into the subcategories “learnability”, “efficiency”, “errors”, and “satisfaction”; each of these subcategories are discussed in turn below.

**Table 4:** Reported usefulness as usability and utility for different functions and features of the PDA

	-------------------------- Utility --------------------------		-------------------------- Usability --------------------------	
Functions	Requested	Used	Evaluated	Comments
Address, phone book	[17,29]^*^	[14,16,21,26,28,43,44,58,59]	[14,16,21,26,28,43,44,58,59]	Valuable and commonly used
Calendar, scheduling	[29]	[16,22,26,28,34,40,43,44,46,47, 49,53,57-59]	[16,22,26,28,34,40,43,44,47,49,57-59]	Commonly used
Memo pads, To Do list	[29]	[16,26,35,43,46,49,59]	[16,35,43,49,59]	Valuable
Internet access, email	[17,28,48]	[14,16,26,28,31,34,44,49,54,59]	[14,16,26,28,34,44,49,59]	Not often used
Phone	[48]	[14,15,26,34,44]	[14,15,34,44]	Improve access
Word processing	[28]	[28,30,40,45,58]	[28,30,40,45,58]	Not often used
Alarm	[17,29,48]	[46]	-	-
Camera	[17,48]	[34]	[34]	Useful
Video	[17]	[33]	[33]	Developable
^*^References refer to publications where the respective function was requested, used or evaluated				

**Table 5:** Reported usefulness as usability and utility for software applications on the PDA

	-------------------------- Utility --------------------------		-------------------------- Usability --------------------------	
Software application	Requested	Used	Evaluated	Comments
Drug information	[17,28,29,39,48]^*^	[16,21,22,24,26,28,30-32,34,35, 38,40,43-45,47,49-51,53-55,57,59,61]	[16,22,24,26,28,30,31,34,35,38, 40,43-45,47,49-51,54,55,57,59,61]	Commonly used
Medical information	[17,28,29,39,48]	[3,14-16,19,24-26,28,30,31,34, 35,38-40,44-46,49-51,53-55,59,61]	[14-16,24,26,28, 30,31,34,35,38-40,44,45,49-51,54,55,59,61]	Commonly used
Guidelines	[16,29]	[37,52,53]	[37,52]	Improve care
Medical list/ orders	[17,29,48]	[25]	[25]	Helpful, reduce error
Medical calculator	[28,29,48]	[16,22,26,28,30,34,38,40,43-47, 49,53,55,58,59,61]	[16,22,26,28,30,34,38,40,43,45,47,49,58,59,61]	Commonly used
Dictionaries	[17,48]	-	-	-
Patient information	[17,28,29,39,48]	[14,16,19,20,25,27,43,44,46,55, 56,58]	[14,16,19,25,43,55]	Useful, conveni­ent
Barcode identification	[48]	[23]	[23]	Reduce human errors
Test results	[17,29,48]	[14,25,51,55]	[14,25,51,55]	Convenient
Prescription	-	[18,19,22,26,40,42,45,47,57]	[18,22,26,40,42,45,47,57]	Increased safety
Billing	-	[22,26,44,46,47,53,57]	[22,26,47,57]	Not often used
Education	-	[19,26,31,35,38,40,41,45,51,53,57]	[35,38,40,41,45,51,53,57]	Preferable
Patient education	[16]	[53,57]	[53]	-
Anatomy atlas	-	[15]	-	
Statistical analysis	-	[20,31,57]	[20,31,57]	Valuable
^*^References refer to publications where the respective software application was requested, used or evaluated				

##### Learnability

The PDA was associated with a fairly high degree of learnability. Practice and support could reduce problems when using a PDA. Some users regarded the tool as easy to understand, while others found it difficult in the beginning. Several technical problems were described, but after guided practice, explanations, and adequate time, many of the problems were solved [20,22,24,31,34,38,41,46]. A majority of residents and faculty reported themselves as self-taught PDA users [49]. To optimize the technology and to overcome barriers, users of PDAs suggested that technical support should always be provided. The users requested that support be available constantly and were aware that there was more they could have accomplished with the PDA if they had sufficient knowledge [30,34,38,43,49,50,57]. There seemed to be a learning threshold at the introductory stage of PDA use. Physicians using a PDA mobile phone device preferred their traditional system, rather than having to learn how to operate a new device. However, after a 6-week trial they found the PDA mobile phone to be user-friendly and its operation easy to learn [15]. Nursing students found the PDA easy to use due to their experience and familiarity with other computers [30]. Many participants had difficulties handling the new and complex hardware and its software applications. They also had difficulty installing software applications and reported a lack of training and time to learn how to operate the PDA [14,49]. Thus, the combination of phone and PDA features may introduce a new degree of complexity for beginners [60].

##### Efficiency

The use of a PDA in health care settings can improve efficiency in many ways, including, for example, decision-making [27,52-55,59]. Its pocket size made the PDA easy to access, and it was considered to be a time-saving device, since it made it immediately possible to find needed information [43,51,57]. Wireless access to the Internet was also considered valuable, since users had a connection everywhere [34]. Second-year nursing students using a PDA loaded with medical software applications felt more confident and effective than peers who did not use a PDA [35]. The PDA can produce positive changes in patients’ care plans [27,51,55], support physicians in medical decisions [53,54], and improve learning for medical students [45], as well as enhance learning for nursing students [51]. Evidence-based guidelines for screening were fast and easy to use at the point of care [52]. The software application of angina diagnosis in a PDA increased the use of cardiac stress-testing by family physicians [37]. Furthermore, having a handheld drug reference guide to find drug information was time-saving [54,57], and the possibility of an immediate search was useful in clinical knowledge deficits [38]. In a case study, participants using a PDA worked faster with a case than the control group [36]. Not everyone agreed that the PDA was time-saving [39,58], but it was believed that using it could lead to more efficient patient care [39]. In general, PDAs were considered to be a convenient tool; on the other hand, the PDA was not believed to decrease paperwork or improve patient health outcomes [50].

##### Errors

Using a PDA can reduce the number of medical errors in health care [18,22,32]. Some physicians felt that they were less likely to lose information when it was collected in their handheld tool, instead of written on paper-based index cards, guideline pamphlets, and calendars [16]. Introducing a barcode system to PDAs for patient identification during a blood transfusion was effective in reducing human errors related to bedside transfusion procedures [23]. Using a PDA-based decision support system in prescribing pharmaceuticals increased safety among PDA using physicians compared to the no-PDA control group [18]. In a case study, the accuracy was higher among nursing students using a PDA than for the control group [36].

##### Satisfaction

Both positive and negative attitudes toward the PDA were reported. The same aspects could be regarded as positive for some of the users and negative for others. The attitudes seemed situation-dependent. Physicians who had used a PDA found it very useful during night duty and in emergency situations, but in doctors’ rounds it was found to be ineffective [14]. Its pocket size was regarded as convenient, as well as the screen size, which was large enough to be clear and easy to read [30,43,51,57]. The speed of getting information is one of its primary advantages [16]. In several studies, the small screen size was mentioned as a barrier to use [22,25,34,41,46,60], as well as its being inconvenient for viewing long documents [14,30,43] and its inability to add marginal notes [41,46].

Patient confidentiality when using a PDA was of no concern compared to using other technologies [50], and physicians had no concern about using the PDA in front of a patient [21]. Nurses and medical students who had used a PDA, both as a reference tool and multimedia technology medium, seemed to value the former in the PDA more than the built-in phone, e-mail, and camera, even though it was convenient to have them in the same tool [34]. The breadth and depth in specially created programs were not always satisfying [40,60]; information was not updated [53]; and a lack of programs was reported for health care specialities such as psychology, orthopaedic and plastic surgery, oncology, and otolaryngology [60]. Some physicians raised a concern about over-reliance on the tool [16,57]. Finally, limited memory and a short battery life were frequently mentioned barriers to use [23,38,40,46,53,57,60]. Nursing students did not find battery life to be a problem as long as they recharged the battery after each shift. To avoid a loss of data through loss of battery power, some students saved their documents to back up files rather than to the main memory [30].

## Discussion

In the present study, we found the PDA to be a valuable tool for personnel and students in health care. The PDA allowed immediate and easy access to medical information that might improve patient care and the quality of health care. We found a number of areas where PDAs were used with different functions and software applications for personnel and students in health care. The main findings were that drug and medical information were accessed most often. We also identified functions that could be added and areas to be improved to take full advantage of the PDA. We hope that this overview of the use of PDAs will provide some direction for future research.

That we ended up with only 48 relevant publications after the quality assessment indicates that few original peer-reviewed research articles have been completed so far. In the articles reviewed, the research approach varied. Most studies were descriptive, and sample sizes and response rates varied. Since PDA intervention studies often entail a small sample size, due to costs and available technical equipment, this might be accepted in our study. This includes one article with a response rate as low as 24% [28], which is a limitation; however, we chose to include that article due to its large sample size. Both the use and the research of PDAs in health care are expanding areas for study which we experienced through our updated literature searches.

The categories which emerged from our content analysis coincided to a certain extent with Nielsen’s Model of System Acceptability [12]. The benefit of using Nielsen’s model as a theoretical framework lies in providing a structure when presenting the results. A limitation of using Nielsen’s model could be the risk of missing significant areas not fitting the model, and we did not cover all the existing categories of the model. However, the model seemed to cover all relevant aspects we found and has been used by others in health care research [62,63].

The various functions and software applications available on a PDA seem to ease the workload for health care personnel and students. Like Baumgart [5], we found that there are numerous medical software applications available for PDAs that can be used in order to improve health care. Since most hospitals are becoming more and more computerized, PDAs seem to be a good complement to stationary computers. It is our belief that to utilize fully its capabilities, the PDA needs to be integrated with hospital networks with access to, for example, patients’ health care records, including patients’ test results and internal memos.

The findings in the present study are not unanimous when it comes to whether or not using a PDA as a tool can save valuable time for personnel and students in health care. Some of the results from the present review are supported by Lu et al [8] who found that PDAs are time-saving for getting immediate access to drug information. Not all users think that a PDA saves time, but PDA users do believe it can deliver faster and more efficient patient care. Thus, an effective use of the tool might imply that more time can be devoted to patients.

The PDA seems to be a feasible and convenient tool, with one of its top advantages being the speed with which one can retrieve information on the spot. Accessibility to updated information can be improved when using a PDA, which provides an opportunity to check for the latest medical information in a convenient way. Access to drug and medical information might improve patient care and make it more effective and, hopefully, time-saving. In the present review, we found that PDAs improve decision-making and point toward positive changes in patient treatment, a conclusion in line with a previous review [5]. The possibility of checking medical orders and patient identification by using, for example, a PDA with a bar-code system, can reduce errors. We are convinced that there is a need for the PDA and that this is a tool for all professionals and students in health care.

Learnability concerns the ease with which one can learn to use a PDA. In the beginning, a PDA might seem to be complex and confusing hardware. To overcome barriers, the challenge is to provide the right support and to create suitable functions and software applications for various health care professionals in various specialities. In accordance with Lu et al [8], we identified several barriers and difficulties when starting to use a PDA. Most of these barriers seem to be more behavioral than technical in nature. To overcome these barriers, guided practice, explanations, and adequate training time are needed, and access to technical support is necessary. Other barriers, such as short battery life and small memory capacity, should be easily overcome by constantly expanding technology. The PDA can also improve learning for students in clinical practice and health care professionals. Participants stated in the Johnson et al study [39] that they learned about new medical developments sooner with a PDA than without one, in which case there might exist medical developments that they had not learned about at all. These important data confirm that a PDA is suitable for both students and professionals to improve learning.

It is difficult to draw definitive conclusions from the studies we reviewed. Altogether, the articles do not represent strong evidence for the benefits of using a PDA. We agree with Berglund et al [17] that a PDA has the potential to be accepted by personnel and students in health care, if the PDA meets their functional and software application needs and is user friendly. To implement fully PDAs in health care, we need more research into functions and software applications. References, mostly from the USA and including physicians and medical students, indicate that several professions are missing from PDA research, including nurses, physiotherapists, and others. Kho et al [9] confirmed that PDAs are appreciated among students, and this is important to explore in future research. Since we noticed similar findings in our own observations, and since students are increasingly requesting PDAs, it is important that functionality and software applications operate smoothly and securely when synchronized with a stationary computer; that the interface is easy to follow; and that patient data is secured. In agreement with Lu et al [8], we note that, to evaluate the effect PDAs have on the quality of medical practice, studies with larger sample sizes are needed. We argue for more research using intervention studies, randomized controlled trials, and action research. Finally, when introducing new technology in health care, there is a need for scientifically based evaluations that take into account not only the technology itself in relation to the individual, but also the organization, including context and costs.

## Abbreviations

CDSS: clinical decision support software
ICT: information communication technology
PC: personal computer
PDA: personal digital assistant
